# A Challenging Case: Botulism in a Toddler

**DOI:** 10.7759/cureus.49924

**Published:** 2023-12-04

**Authors:** Carolina Castro, Joana Machado Morais, Ana Luisa Correia, Rui Almeida, Sérgia Soares

**Affiliations:** 1 Pediatrics, Hospital Pedro Hispano, Matosinhos, PRT

**Keywords:** paralysis, ptosis, botulinum neurotoxin, clostridium botulinum, foodborne botulism

## Abstract

Botulism is a life-threatening, rapidly progressive neuroparalytic disease caused by one of the most potent toxins known, botulinum toxin. It manifests as flaccid and symmetrical descending paralysis that can affect both cranial and peripheral nerves. The only specific treatment available is the administration of botulinum antitoxin. We present the case of a three-year-old boy who had gastrointestinal symptoms and had ingested garden soil/dust at a construction site before the onset of cranial nerve palsy, which manifested as dysphagia in swallowing liquid and solid food and bilateral progressive ptosis. Early suspicion of botulism and treatment with botulinum antitoxin resulted in complete neurologic recovery. This case highlights the importance of a careful history and neurologic examination to avoid misdiagnosis. Administration of botulinum antitoxin should not be delayed until the diagnosis is confirmed and clinicians should be aware that this approach can be life-saving.

## Introduction

Botulism is a rare and potentially fatal syndrome of diffuse flaccid paralysis caused by neurotoxins produced by some *Clostridia species*, most commonly *Clostridium botulinum* [1]. *Clostridium botulinum* is a spore-forming, obligate, anaerobic, gram-positive bacillus widely distributed in the environment [2,3]. Botulinum neurotoxins (BoNTs) are considered one of the most potent toxins known to target motor neurons; they act by blocking the cholinergic innervation of muscles in various tissues [2,3].

Different circumstances of toxin propagation can lead to the four main forms of human botulism: infant, foodborne, wound, and adult intestinal botulism. There are also rare case reports of inhalational and iatrogenic botulism following cosmetic or therapeutic injection of BoNTs [3]. The clinical syndrome of human botulism is characterized by acute, progressive, symmetric, and descending muscle weakness with early cranial nerve involvement [4].

We describe the case of a three-year-old boy in whom early suspicion of botulism based on clinical and epidemiologic findings allowed prompt administration of botulinum antitoxin, which contributed to a favorable outcome. This article was previously presented as a poster at the Paediatric Infectious Diseases Congress on May 8, 2023.

## Case presentation

A three-year-old boy with developmental delay was admitted to the emergency department with decreased spontaneous activity, dysphagia in swallowing liquid and solid food, and bilateral progressive ptosis of the eyelids for 24 hours. The parents reported vomiting and copious diarrhea the day before admission and consumption of garden soil/dust at a construction site a week before the onset of symptoms. Consumption of honey, homemade canned foods, or unpasteurized dairy products was denied. No other epidemiologic link was found.

**Figure 1:**
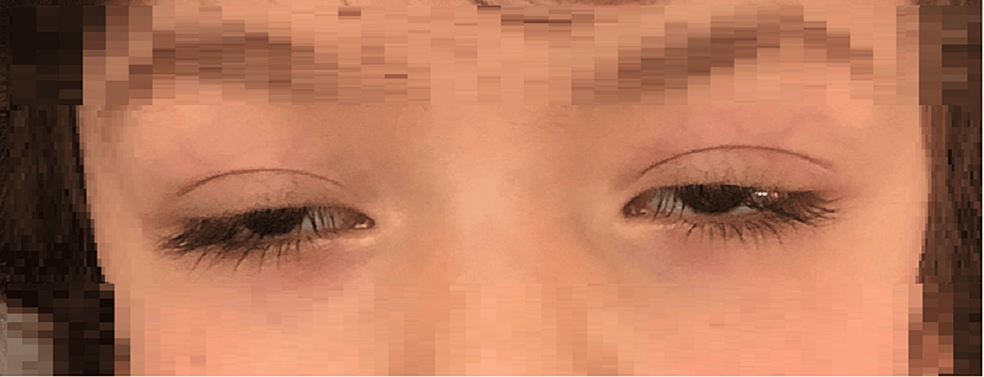
Bilateral ptosis on the first day of hospitalization

On arrival, the child was prostrated, without hemodynamic instability or fever. Physical examination also showed sialorrhea. Neurologic examination revealed bilateral ptosis of the eyelids (Figure 1), normal symmetrical pupils with direct and consensual response to light, and no limitations of ocular motility or facial asymmetries. No other signs of muscle weakness or dysautonomia were noted, and osteotendinous reflexes were present and symmetrical. The basic metabolic panel, complete blood count, and C-reactive protein were within normal limits, and the urine test for drugs was negative. The child was admitted for monitoring and etiologic evaluation of this sudden-onset neurologic disorder.

During hospitalization, he remained apyretic, and the deficits persisted without respiratory impairment or progression of muscle weakness. Later, the patient also presented constipation. Magnetic resonance imaging of the brain was normal, and a negative anti-acetylcholine receptor antibody and anti-acetylcholine receptor-associated protein antibody ruled out Myasthenia Gravis (MG) as the cause of the deficits.

On the second day, when the deficits persisted without apparent cause, the hypothesis of botulism was raised in view of the epidemiological context (ingestion of soil/dust) and the rapid clinical evolution. Serum, gastric aspirate, and stool samples were obtained, and equine heptavalent botulinum antitoxin was administered on the third day of hospitalization. The patient evolved positively after antitoxin administration and showed gradual improvement in eating problems and ptosis.

Although laboratory results did not show evidence of botulinum toxin, a clinical diagnosis of botulism was made because the neurological deficits improved after specific therapy. The case was reported to the National Public Health Services. The patient was discharged home after eight days of hospitalization. At that time, he had neither eating problems nor sialorrhea and showed mild spontaneous eye opening on the left side (the child raised the right eyelid with his hand to reach for objects). Neuropediatric follow-up confirmed complete recovery without sequelae after five months.

## Discussion

Botulism is an almost forgotten disease that can appear suddenly without arousing suspicion. It can be misdiagnosed as Guillain-Barré syndrome (GBS) or MG. Urgent treatment is needed to prevent disease progression, persistent deficits, or death [5]. Our case report illustrates the pitfalls of this diagnosis. Because exposure to the preformed toxin most likely occurred through consumption of contaminated soil, this case can be classified as foodborne botulism. Most cases of foodborne botulism occur sporadically, and outbreaks are usually small. However, it remains a public health concern. All suspected cases must be reported so that public health authorities can identify risk factors for intoxication and prevent further cases [6,7].

*Clostridium botulinum* spores are ubiquitous in the environment and can survive most naturally occurring conditions as well as boiling. When BoNTs enter the bloodstream (through ingestion via food or dust, inhalation, injection, colonized wounds, or the intestine), they are transported to the peripheral cholinergic nerve endings of autonomic and voluntary motor muscles and irreversibly bind to cholinergic receptors of the presynaptic cell membrane [6,8].

The signs and symptoms of botulism may develop and progress within a few hours to several days. In foodborne botulism, early gastrointestinal symptoms (nausea, vomiting, abdominal pain, diarrhea) are common and usually precede neurologic symptoms, as observed in this case. The head, face, and neck muscles are also affected early in the course of the disease, resulting in bulbar palsy characterized by dysphagia, decreased gag reflex, drooling, ptosis, and facial weakness. The extent of neurologic symptoms may range from isolated ptosis or mild cranial nerve involvement to descending bilateral paralysis that may ultimately affect the diaphragm and respiratory muscles and cause respiratory failure if more than 90% of the receptors are blocked [5,6,8]. In some advanced cases, decreased heart rate variability or other autonomic disturbances may occur. BoNTs do not cross the blood-brain barrier, so cognitive function and sensorium are not affected [1]. The main complication reported is hypoxic brain injury following respiratory arrest [8].

On diagnostic workups, laboratory and imaging tests are usually unremarkable. Nerve conduction studies and electromyography can be extremely useful in differentiating botulism from atypical cases of GBS in adults and other unclear cases. They are rarely used in children because they are painful and not diagnostic despite the characteristic pattern [1,8]. Laboratory confirmation is possible by detecting BoNTs in clinical or food samples. Early collection of specimens (serum, stool, and/or gastric contents) before antitoxin administration may increase the likelihood of obtaining case confirmation [9]. In this particular case, the fact that the specimens were collected on the fourth day after the onset of neurologic symptoms may have influenced the result.

Common misdiagnoses on admission include sepsis, dehydration, drug side effects, and viral syndromes. Other differential diagnoses to consider are MG, GBS and its variant Miller-Fisher syndrome, atypical tetanus, diphtheria, and poliomyelitis [5,8]. Currently, equine botulinum antitoxin is the first choice for the treatment of botulism (except for cases of infant botulism) because of its availability, efficacy, and safety. Early administration of antitoxin can reduce the extent and severity of paralysis and, in some cases, prevent respiratory paralysis or shorten the duration of mechanical support and ICU stay. Because the binding of BoNTs to nerve endings is irreversible, neurologic recovery occurs over weeks to months as motor neurons regenerate [1,10]. As we saw in this case, full recovery occurred only after five months. Antibiotics are not recommended because of the hypothetical risk of increasing the amount of toxin in the colon and worsening the patient’s clinical condition. The only indications for antibiotic use are secondary bacterial infections such as pneumonia, urinary tract infections, and otitis media [10].

Although botulism is an “old” and well-described disease, from a clinical point of view, it must be emphasized that it is still a rare intoxication characterized by difficult clinical suspicion [9]. Diagnosis depends on strong clinical suspicion and careful neurologic examination. Timely diagnosis is crucial for successful treatment because the only specific treatment available, botulinum antitoxin, needs to be administered as soon as possible [1,6].

## Conclusions

The key element in this case was the high index of suspicion for this rare entity. A careful history and thorough neurologic examination are essential for the diagnosis of botulism, which can be made on the basis of clinical and epidemiologic findings. Early administration of botulinum antitoxin neutralizes the free neurotoxin and should not be delayed. This approach may reduce the severity, duration, and mortality of this disease.
